# Embodied decisions during walking

**DOI:** 10.1152/jn.00149.2022

**Published:** 2022-10-05

**Authors:** Eric Grießbach, Philipp Raßbach, Oliver Herbort, Rouwen Cañal-Bruland

**Affiliations:** ^1^Department for the Psychology of Human Movement and Sport, Friedrich Schiller University Jena, Jena, Germany; ^2^Department of Psychology, Julius-Maximilians-Universität Würzburg, Würzburg, Germany

**Keywords:** decision-making, embodied choices, gait, motor cost, reward

## Abstract

Research on embodied decision-making only recently started to examine whether and how concurrent actions influence value-based decisions. For instance, during walking humans preferably make decisions that align with a turn toward the side of their current swing leg, sometimes resulting in unfavorable choices (e.g., less reward). It is suggested that concurrent movements influence decision-making by coincidental changes in motor costs. If this is true, systematic manipulations of motor costs should bias decisions. To test this, participants had to accumulate rewards (i.e., points) by walking and turning toward left and right targets displaying rewards across three experiments. In *experiments 1a* and *1b*, we manipulated the turning cost based on the current swing leg by applying different symmetric turning magnitudes (i.e., same angles for left and right targets). In *experiment 2*, we manipulated the turning cost by administering asymmetric turning magnitudes (i.e., different angles for left and right targets). Finally, in *experiment 3*, we increased the cost of walking by adding ankle weights. Altogether, the experiments support the claim that differences in motor costs influenced participants’ decisions: *experiments 1a* and *1b* revealed that the swing leg effect and stepping behavior were moderated by turning magnitude. In *experiment 2*, participants showed a preference for less costly, smaller turning magnitudes. *Experiment 3* replicated the swing leg effect when motor costs were increased by means of ankle weights. In conclusion, these findings provide further evidence that value-based decisions during ongoing actions seem to be influenced by dynamically changing motor costs, thereby supporting the concept of “embodied decision-making.”

**NEW & NOTEWORTHY** Motor processes of concurrent movements have been shown to influence embodied decision-making. It is hypothesized that this is driven by coincidental changes in motor costs. We tested this claim by systematically manipulating motor costs of choice options during walking. In three experiments we show how variations in motor cost (e.g., turning angle or stepping constraints) bias decision-making, thereby supporting the concept of “embodied decision-making.”

## Graphical abstract

**Figure:**
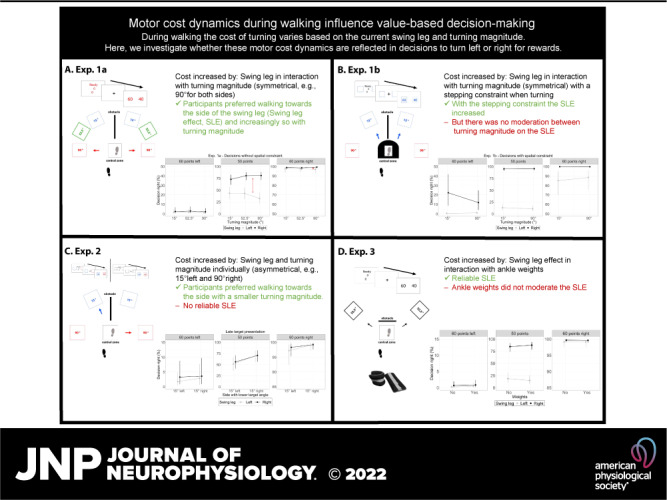

## INTRODUCTION

Imagine being on a soccer field. You dribble the ball while approaching a defender. What will you do next? Should you try to get past the defender on the left or the right, or should you even pass the ball to one of your teammates?

As emphasized by embodied decision accounts, the decision is not merely driven, for instance, by the question of how big the expected values of rewards are (e.g., whether passing the defender on the left or right gets you in a better position to score) but also by the motor costs it takes to turn left or right (1, 2). Obviously, if the defender is positioned further to the left, you may not choose to turn to the left side but turn toward the right side. However, while approaching the defender your position in regard to the defender constantly changes. Accordingly, if the angle to turn to one side increases/decreases, so do the motor costs (3, 4), which may further discourage/encourage the choice to turn to that particular side. This example aims to illustrate that the decision to achieve a goal may be influenced by concurrent movements and concomitant changes in motor costs, a corollary put forth by advocates of embodied decision models (5, 6).

More specifically, action-based models of embodied decisions, like the embodied choice framework (5) or the affordance competition hypothesis (6), argue that action and decision-making can mutually influence each other, hence not only allowing decision-making processes to command certain actions but also allowing action requirements that change as a function of time (action dynamics) to modulate decision-making processes. In contrast to classical decision-making models, which assume independent and sequential processing stages of decision-making and action (i.e., decisions are formulated as an abstract value comparison process that is independent of action, and action only starts after a decision has been made; see Refs. 7, 8), embodied decision models assume that actions influence decisions by parallel state-dependent feedback (5, 8, 9) or more directly that the decision process itself takes place as a biased competition between action representations (6, 8). Especially the latter case blurs the line between decision-making and action.

From an empirical perspective, embodied decisions during ongoing movement have only recently started to be put under experimental scrutiny (10, 11). The few existing studies have thus far mainly focused on the dynamic motor cost in decision-making (12–15). For example, Nashed, Crevecoeur, and Scott (12) asked participants to reach toward one of multiple lateral targets in their experiment 1b. While participants were reaching toward a chosen target, a noticeable lateral force was applied to the hand, displacing it from the reaching direction. Dependent on the strength of the displacement, participants rerouted their hand movement toward a now more suitable target. That is, a dynamic change of the body state, which affected the motor costs associated with each target, determined the selected target.

**Figure 1.**
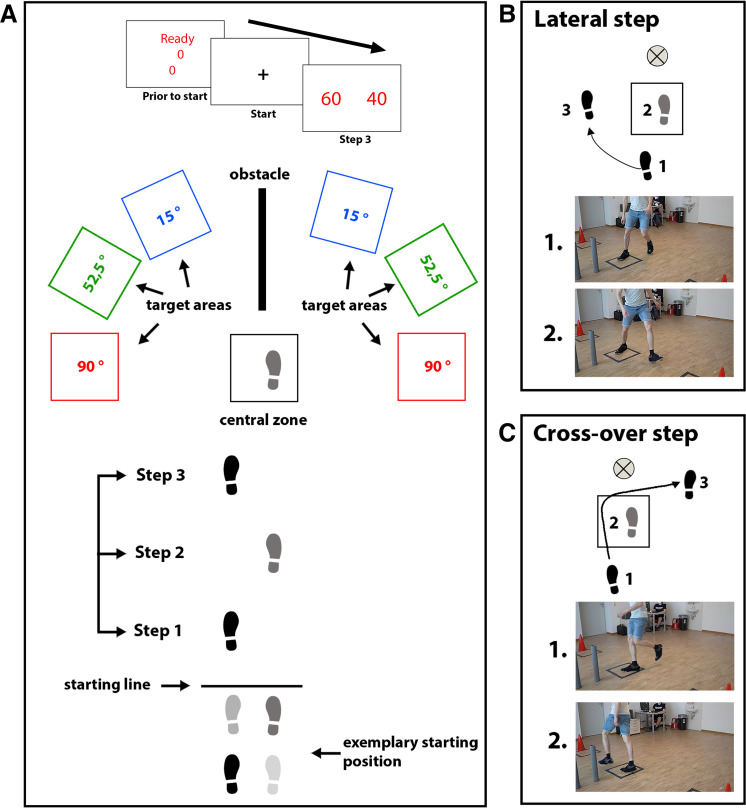
Experimental setup of *experiment 1a*. *A*: participants started by positioning their feet in the required starting position. The projection at *top* shows the time course of cues. The starting position was displayed with a projector on the opposite side of the room (see *top*). The font color represented available lateral targets to finish a trial (here red, representing 90° targets). The German word “Bereit” was used in the experiment instead of “Ready” as displayed here. After participants took the required starting position, a “+” appeared as the Go signal and they walked toward the central zone. After the third step, rewards appeared on the left and right sides of the screen. Participants had to step into the central zone and walk toward a target area to finish a trial and receive rewards. *B*: example of a lateral step. Given that the right foot stepped into the zone and participants chose to walk to the left side, a lateral step can be taken. *C*: example of a crossover stepping strategy. Given that the right foot stepped into the zone, we assumed that participants make a crossover step toward the right side.

This finding provided initial evidence for the influence of action dynamics on motor decisions. However, it remained to be determined whether action dynamics influence decisions involving reward differences (e.g., the expected value of decisions in soccer, see introductory example; see also Ref. 16). To address this issue, Grießbach et al. (14) aimed to analyze the influence of the dynamic motor costs during walking on reward-based decisions (for reaching, see also Refs. 13, 17). More specifically, during walking the alternating swing leg influences the motor cost of turning (18–20). A turn toward the side of the swing leg enables the participant to place the next step lateral to the side of the stance leg (e.g., left swing leg and a leftward turn, hereafter referred to as “lateral step”; see Fig. 1*B*). A change opposite to the swing leg requires crossing the stance leg (e.g., left swing leg and rightward turn, hereafter referred to as “crossover step”; see Fig. 1*C*). People generally prefer to turn toward the side enabling a lateral step (21, 22). To investigate whether and when the concurrent movement of walking influences reward-based decisions (14), participants were instructed to walk toward a central obstacle and then bypass it to collect rewards at a left or right target (for a similar setup, see Fig. 1*A*). Before turning toward the left or right target, they had to step into a central zone in front of the obstacle. Rewards were displayed at various time points during walking. Results showed that participants preferred walking toward the side enabling a lateral step based on the current swing leg (hereafter referred to as “swing leg effect”). Independent of when the rewards were displayed (early vs. late), there was a preference to walk toward the side enabling a lateral step, even to the degree that fewer rewards were obtained. The preference for the lateral step indicated that the anticipated dynamic motor costs for whole body movements like walking influence value-based decisions.

**Figure 2.**
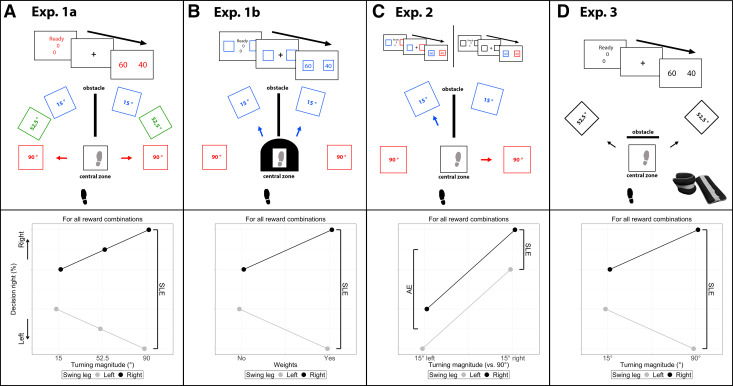
Experimental setup and hypothesis plots for all experiments. *A*: *experiment 1a* displays the basic design used for the rest of the experiments. *Bottom*: the hypothesis of *experiment 1a*: we expected the swing leg effect (SLE) to increase with turning magnitude. *B*: based on the results of *experiment 1a*, in *experiment 1b* a stepping constraint was placed with carpet on the central zone. Additionally, only the 15° and 90° target angles were included and the cue for targets changed to rectangles on the sides (here blue for 15° turning magnitude). For *experiment 1b* we had the same hypothesis as in *experiment 1a*, pictured at *bottom*. *C*: in *experiment 2* the target angle could be asymmetric (here left 15° in blue and right 90° in red). The target angles were displayed either before participants started walking (*top left*, control condition) or with rewards in the third step (*top right*). *Bottom*: the hypothesis of *experiment 2*: we expected for both the early and more importantly the late target timing a preference to walk toward the side with a smaller turning magnitude (angle effect, AE), in addition to the SLE. *D*: In *experiment 3* participants walked with and without ankle weights. Additionally, only 52.5° turning magnitude and the same obstacle as in a prior study (14) were used instead of the cones. *Bottom*: the hypothesis of *experiment 3*: we expected the weights to increase the SLE.

Although Grießbach et al. (14) as well as others (12, 13) argue that concurrent movement influences decision-making by coincidental changes in motor costs, more recent findings (15) indicate that concurrent movement could influence decision-making also by means of shared cognitive representations (e.g., spatial representations such as left, right, top, and down) causing cognitive cross talk, and not necessarily by the motor costs alone. This idea originates from findings of multitasking research where an action in one of two independent tasks can bias responses in the second task if both tasks dimensionally overlap (23, 24). Thus far, however, cognitive cross talk and motor cost dynamics have not, with very few exceptions (15, 25, 26), been differentiated experimentally in embodied decisions. Notably, it cannot be ruled out that the swing leg effect found in Ref. 14 may have been driven by shared representations (i.e., cognitive cross talk resulting from the overlap between the mental representation of the swing leg and decision-making on the left-right dimension) rather than by action cost dynamics. Therefore, the present study aimed to examine the influence of cost dynamics independent of cognitive cross talk on decision-making by manipulating action costs of embodied decisions during walking. To this end, we extended the walking paradigm of Ref. 14 by holding the concurrent movement (i.e., the swing leg) during decision-making constant and systematically manipulating the motor costs associated with each reward option. If the cost dynamics while walking influence participants’ decisions, systematic manipulations of motor costs should be reflected in more or less biased decisions (see Fig. 2).

One factor influencing costs while walking concerns the turning magnitude. First, for symmetric differences between left and right turns (e.g., 15° left and 15° right vs. 90° left and 90° right), the cost difference between a lateral step and a crossover step increases with turning magnitude (19). Hence, we expected the swing leg effect to be moderated by symmetrically increased turning magnitudes (see *experiment 1a* and *experiment 1b*). Second, as smaller turns are less costly (3, 4), we expected participants to prefer smaller turns while walking toward an asymmetric intersection (e.g., 15° left and 90° right; see *experiment 2*). Third, we manipulated the motor costs by administering ankle weights, expecting the swing leg effect to increase (see *experiment 3*).

## METHODS

### *Experiment 1a*: Is the Swing Leg Effect Moderated by Turning Magnitude?

The crossover step is less stable and requires more muscular demands compared to a lateral step. One reason for the higher motor costs for crossover steps is that the center of mass must be placed outside the base of support of the foot for a short time (18, 19). The displacement of the step outside the base of support increases with turning magnitudes for the crossover step, which is not the case for the lateral step. Indeed, the crossover step shows increasing motor demands for a 90° turn compared to straight walking, whereas the lateral step does not (19). With the assumption that a crossover step approaches the energetic demands of straight walking when the turning magnitude decreases, it should become less difficult to make a turn with a crossover step versus a lateral step for smaller turning magnitudes.

Therefore, in *experiment 1a* we focused on the moderation of the swing leg effect by the required turning magnitude. We manipulated the angle between the left and right lateral targets symmetrically between trials (15°/52.5°/90° left and 15°/52.5°/90° right). The required turning magnitude was displayed before starting a trial. We hypothesized that participants would be biased to walk toward the side of the swing leg to avoid the crossover step. Additionally, if motor costs are responsible for the swing leg effect, we would expect an increasingly stronger swing leg effect with increasing turning magnitudes, 15°, 52.5°, and 90°, respectively.

#### Participants.

Based on previous studies (10, 11), we aimed for a sample size of 36 participants in the final analysis. For *experiment 1a*, we recruited 45 participants from local universities. To ensure a predictable timing of rewards and swing leg when changing direction, the experiment required taking four steps before stepping into the zone, unbeknownst to our participants. Note that we chose not to explicitly prescribe four steps because such a prescription might have caused participants to change their spontaneous (i.e., natural/usual) walking behavior, which in turn would diminish the ecological validity of our experiment. Five participants were excluded because they frequently violated the criterion of making four steps (>59%). This resulted in a final sample size of *n* = 40 (mean age 24.6 yr, SD = 3.4; 23 females, 17 males; 38 right-handed, 2 missing data for handedness; 34 right-footed, 3 no preference, 2 left-footed). Participants received €10.00 compensation after the experiment, independent of performance. All participants gave informed written consent before starting the experiment. All experiments in the study were part of a research program that was approved by the ethics committee of the Faculty of Social and Behavioral Sciences of the Friedrich Schiller University Jena (FSV 19/04).

#### Apparatus and stimuli.

The experiment took place in a 5.90-m-long room (Fig. 1). The maximal distance from the starting line to the center of the central zone was 3.41 m. Black tape was used to mark the starting line, the central zone, and the lateral targets. All targets were 0.5 m × 0.5 m and in an arc with a 1.5-m distance from the central zone (from center to center). Colored cones (red, green, and blue) behind the lateral targets were used to mark the target areas and required turning magnitude for a given trial (15°, 52.5°, or 90°).

To prevent participants from switching sides after the central zone, three pipes (radius = 3.7 cm, height = 55 cm) were placed as obstacles separating the left and right sides after the central zone (60 cm behind the center of the central zone and with 30 cm distance between obstacles).

Stimuli were presented with a digital projector (NEC Corp., Tokyo, Japan; model M353WS, WXGA resolution, 60-Hz frame rate) placed on the ceiling over the central zone, projecting on a large screen facing the participant (2.92 m width × 1.83 m height). The screen was 1.80 m behind the center of the central zone. Stimuli were presented on a white background and could be either green, blue, or red, respectively (see Fig. 1). All stimuli were presented with a self-written script in MATLAB in real time based on the kinematic measurements (see *Data analysis*).

Gait behavior was recorded by a three-dimensional (3-D) infrared system (Prime 17 W; OptiTrack, Corvallis, OR) with 12 cameras (120 Hz) and passive reflective markers (12 mm) placed on the lateral malleolus and heel and between the second and third metatarsal head of both feet.

Starting position and the time constraint were scaled individually for each participant (Supplemental Material; see endnote). Participants received auditory feedback indicating whether they finished in time after each trial. Auditory feedback was a beep (750 Hz for 0.8 s) or a double beep (750 Hz, 2 times for 0.3 s with 0.2-s pause between) with the integrated speaker of the projector and a sampling rate of 48,000 Hz. The meaning of the beep and double beep (in time or too late) was counterbalanced between participants.

#### Procedure.

After participants provided informed consent and filled out a demographic data questionnaire, the instructor attached reflective markers on the lower extremities. The experiment started with five calibration trials. Next, participants watched a narrated presentation of the instruction. Their task was to collect rewards by walking toward one of two lateral targets displayed by the color of the projected stimuli.

Participants were prompted to position their feet into the predetermined starting position (left or right foot in front at the starting line) to start a trial. When the feet maintained the position for 1.5 s the prompt for the starting position was replaced with a central “+” as the Go signal. At the third step, rewards for left and right targets were displayed (participants had not been informed about the exact timing). As rewards, one of three point combinations could be displayed (left/right: 40/60, 50/50, or 60/40). Before walking toward a lateral target, participants had to step into the central zone. To finish a trial, participants had to change direction to step with both feet into one of the two relevant lateral targets. After the trial, a sound signaled whether participants were in time. If participants were in time (see Supplemental Information for the determination of the time constraint), they got the reward for the side of the target they finished. If participants were not in time, they received the lower reward (40 points if 60/40, 50 points if 50/50). After finishing a trial, participants walked back to the starting line and positioned their feet to start the next trial.

Each participant completed a total of 18 familiarization trials and 168 experimental trials. The experimental phase included 2 (starting position: left/right foot at starting line) × 3 (turning magnitude left/right: 15°/15°, 52.5°/52.5°, 90°/90°) × 3 (point combination left/right: 40/60, 50/50, 60/40) × 14 trials for the equal rewards (50/50) and × 7 trials each for unequal rewards (60/40 and 40/60) so that equal and unequal rewards were presented in the same number of trials. Trials were presented in random order. The experiment lasted ∼70 min.

#### Data analysis.

##### Online analysis.

Stimuli were presented in real time based on participants’ kinematics of the tracked marker. The 3-D positions of markers were streamed with the NatNet SDK from Motive v2.1.1 (OptiTrack software interface) to a self-written MATLAB 2018a script (The MathWorks, Inc., Natick, MA). We determined the start of a trial, the timing of reward presentation at the third step (27), the step in the central zone, and the end of a trial based on the positioning of the foot markers (see Supplemental Material).

##### Offline analysis.

For further analysis and validation of real-time data of experimental trials, kinematic data were filtered at 12 Hz with a bidirectional fourth-order low-pass Butterworth filter. We interpolated missing values up to 25 frames (0.21 s, cubic spline interpolation). We checked individual kinematic data of suspicious trials visually (see Supplemental Information for detection methods). After visual inspection of these trials, 6,443/7,560 trials (87.9%) were included in the statistical analysis. The remaining 12.1% of trials were predominantly excluded because participants made three or five steps instead of four (1,034 trials) and some trials because of various problems with the instruction or measurement (83 trials, see Supplemental Information for specifics).

For statistical analysis, we used R (28). To investigate the influence of swing leg (left or right), reward combination (60/40, 50/50, and 40/60 for the left/right side), and turning magnitude (15°/15°, 52.5°/52.5°, 90°/90° left/right) on participants’ decisions (left/right side) we used the Bayes version of a generalized linear mixed model (26). We assumed a Bernoulli distribution for the outcome variable decisions (left or right) and used a logit link function. Model fitting was done with the brms package (29). We followed the guidelines of Ref. 30. Our scripts can be found at https://doi.org/10.17605/OSF.IO/C8MUS.

We used a priori-specified contrasts based on our hypothesis (31). The factor reward was included as a Helmert contrast to investigate a side difference when the reward combination was unequal (40 points right vs. 60 points right) and to compare the effects of unequal rewards with the equal reward combination (mean of 40 points right and 60 points right vs. 50 points right). For the turning magnitude, we used a sliding difference contrast to investigate effect differences from 15° versus 52.5° and 52.5° versus 90°. For the swing leg, we used a centered sum contrast to compare the effect of the right swing leg (−0.5) versus the left swing leg (+0.5). We did include a random intercept and all random slopes as random effects for subjects (32), but we excluded correlation parameters between random effects as they do not influence estimations of fixed effects but increase model complexity and the resulting computation time (33). The priors are specified in the Supplemental Information. The formula for the model in the R script reads

$$\;\text{logit}\left({\text{p}}_{\text{Side}}\right)∼\text{Points}\_\text{R}*\text{Swing}\_\text{Leg}*\text{Turning}\_\text{Magnitude}+\left(\text{Points}\_\text{R}*\text{Swing}\_\text{Leg}*\text{Turning}\_\text{Magnitude}||\text{Subject}\right)$$

For each parameter, the Bayesian model provides a posterior distribution. The posterior distribution is a probabilistic representation of parameter values given the priors, the likelihood of the data, and the model. To summarize the posterior distribution, we provide the exponentiated estimated mean [exp($\hat{\beta }$), odds ratio (OR)], the corresponding 95% credible intervals (CrIs) (equal-tailed intervals), and the probability for samples below or over a certain value. The 95% credible interval defines the range within which the parameter value falls with a probability of 95%. We highlight parameters that are highly probable to be greater or smaller than a null effect (>95%) below in the text. If not otherwise specified, this was the approach for all other Bernoulli-distributed Bayesian mixed models.

### *Experiment 1b*: Replication of *Experiment 1a* with a Stepping Constraint

To prevent the transition step in *experiment 1b*, we shrank the central zone to provide only enough space for a single foot and surrounded it with a no-step zone (marked by a carpet). Additionally, we used only the 15° and 90° turning magnitudes. Again, we hypothesized that the spatial constraint would increase the number of crossover steps when walking toward the opposite side of the swing leg and thereby increase the moderation of the swing leg effect by turning magnitude, resulting in a small and a large effect for the 15° and 90° conditions, respectively.

#### Participants.

Forty-three participants were recruited, five of whom were excluded because of the four-step criterion (>51.0%). This resulted in a sample size of *n* = 38 (mean age 23.9 yr, SD = 3.2; 21 females, 17 males; 34 right-handed, 3 left-handed). All participants received a performance-independent compensation of €10.00 and gave informed consent before starting the experiment.

#### Apparatus and stimuli.

The setup of *experiment 1b* was almost identical to that of *experiment 1a*. In contrast to *experiment 1a*, we only used 15° and 90° targets, displayed by red or blue cones. Additionally, we aimed to constrain the transition step by reducing the size of the central zone and putting a black semicircular carpet around the zone based on the positioning of transition steps in *experiment 1a* (see Fig. 4*D*, central zone: length = 0.35 m, width = 0.2 m, carpet: 0.5 m radius, 0.15 m distance before central zone, see Supplemental Video).

#### Procedure.

The procedure was almost identical to *experiment 1a*. If participants stepped onto the carpet, the instructor repeated the instruction to not step onto the carpet. Each participant completed a total of 12 familiarization trials and 96 experimental trials. Experimental trials included 2 (starting position: left/right foot at starting line) × 2 (turning magnitude left/right: 15°/15°, 90°/90°) × 3 (point combination left/right: 40/60, 50/50, 60/40) × 12 trials for the equal rewards (50/50) and × 6 trials each for unequal rewards (60/40 and 40/60) so that equal and unequal rewards were presented in the same number of trials. The trial order was randomized. The experiment lasted ∼50 min.

#### Data analysis.

We used the same online and offline analysis as in *experiment 1a*. After visual inspection, 3,332/4,128 trials (80.7%) were included in the statistical analysis. The remaining 19.3% of trials were predominantly excluded because participants made three or five steps instead of four (742 trials) and some trials because of various problems with the instruction or measurement (54 trials; see Supplemental Information for specifics). We used almost the identical statistical modeling approach as for *experiment 1a*. However, as there were only two levels for the turning magnitude, the angle was now coded as a centered sum contrast (−0.5 for 15° and +0.5 for 90°).

### *Experiment 2*: Turning Magnitude Influences Decision-Making

In *experiment 2*, we manipulated the motor cost differences for left and right turns by administering asymmetric turning magnitudes (i.e., different angles for left and right targets) because the turning magnitude relates to the energetic demands of walking independent of the swing leg (3, 4). In short, a 15° turn is energetically less costly compared to a 90° turn. Additionally, angle influences motor decisions concurrent to actions in reaching tasks (26, 34). For example, target selection while reaching is biased to targets that are aligned to the concurrent reaching movement (e.g., 30°) compared to less aligned targets (e.g., 90°, Ref. 26).

To investigate whether cost differences by means of the turning magnitude are part of the decision process, turning magnitudes were asymmetrically presented between both choice options in *experiment 2* (e.g., 15° left and 90° right). To test whether motor costs resulting from the target angle could also be considered during action execution, the required turning magnitudes were presented while participants were walking. As a control condition, we provided information about the turning magnitude before participants started walking. *1*) If the required motor costs of turning influence decision-making, we expected participants to preferably walk toward targets with a smaller turning magnitude (15° target) compared to a larger turning magnitude (90° target) for both presentation timings of the turning magnitude. *2*) Additionally, as the motor costs change for both the turning magnitude and the swing leg, we also expected the decision to be influenced by both individually.

#### Participants.

Forty-three participants from local universities were recruited, four of whom were excluded for violating the four-step criterion. This resulted in a sample size of *n* = 39 (mean age 23.6 yr, SD = 3.8; 24 females, 15 males; 34 right-handed, 3 no hand preference, 2 left-handed). All participants received €15.00 compensation and gave informed consent before starting the experiment.

#### Apparatus and stimuli.

The same apparatus and stimuli were used as in *experiment 1a*. Identical to *experiment 1b*, we only used 15° and 90° targets. Again, the color on the screen represented the target option in each trial. The display of the targets changed compared to *experiment 1*. The targets were now displayed by rectangles on the left and right sides of the screen and could have the same or different colors, meaning that the required turning magnitude for the left and right sides could be the same or different. For example, for asymmetric angles, the left rectangle could be blue and the right one red, indicating that participants had to finish at the 15° left target or at the 90° right target (see Fig. 1). The colors for the turning magnitude were displayed before the trial or with the third touchdown when displaying the reward combinations.

#### Procedure.

The procedure was almost identical to *experiment 1a*. One difference was that participants were instructed that asymmetric and symmetric angle combinations could occur and that turning magnitude was presented before starting a trial (early target presentation) or concurrent with walking toward the obstacle (late target presentation). The presentation timing for turning magnitudes was manipulated blockwise. Each participant completed a total of 12 familiarization trials and 96 trials per timing block, in sum 24 familiarization trials and 192 experimental trials. Per presentation timing block, experimental trials included 2 (starting position: left/right foot at starting line) × 4 (target combination left/right: 15°/15°, 90°/90°, 15°/90°, 90°/15°) × 3 (point combination left/right: 40/60, 50/50, 60/40) with 2–8 trials dependent on the condition. The number of trials was not balanced, with fewer trials in the symmetric turning magnitude condition (2 trials for unequal rewards and 4 trials for equal rewards vs. 4 trials for unequal rewards and 8 trials for equal rewards). The trial order was randomized. The order of experimental block (timing of lateral targets) was counterbalanced between participants. Between blocks, participants had a 2-min pause to rest in a chair. The experiment lasted ∼75 min.

#### Data analysis.

We used the same online and offline analysis as in *experiment 1b*. After visual inspection, 6,768/8,256 trials (82.0%) were included in the statistical analysis. The remaining 18.0% of trials were predominantly excluded because participants made three or five steps instead of four (1,347 trials) and some trials because of various problems with the instruction or measurement (141 trials; see Supplemental Information for specifics).

To reduce the complexity of the model, we focused our main analysis on the comparison of asymmetric turning magnitudes and decided to exclude trials with symmetric turning magnitudes. The respective model included starting position (left and right), reward combination (40/60, 50/50, and 60/40), target combination (15°/90° and 90°/15°), and timing of target presentation (early and late) as independent variables and participants’ decisions (left/right) as the dependent variable. Contrasts and priors were identical to *experiment 1b*. Additionally, target timing was included as a centered sum contrast. We included all random effects terms (slopes and intercept) without correlations between the random effects. The formula for the model in the R script reads

$$\text{logit}\left({\text{p}}_{\text{Side}}\right)∼\text{ Target}\_\text{Timing}*\text{Points}\_\text{R}*\text{Swing}\_\text{Leg}*\text{Turning}\_\text{Magnitude}+\left(\text{Target}\_\text{Timing}*\text{Points}\_\text{R}*\text{Swing}\_\text{Leg}*\text{Turning}\_\text{Magnitude}||\text{Subject}\right)$$

### *Experiment 3*: Swing Leg on Decision-Making with Ankle Weights

Increasing the weights of the legs increases the energetic demands and generally decreases the stability of walking, presumably independent of the current swing leg (35–37). The association between motor costs and decision-making is found to be nonlinear in sequential decision-making: There is a parabolic relationship between motor effort and participants’ decisions (2), and for stability there exists a boundary of how much perturbation is tolerable before falling or not falling (38). This nonlinear relationship results from the maximum force the muscles can generate and a buffer to tolerate perturbation before falling, making more extreme values even less preferable. Given that the crossover step is less stable and more demanding compared to the lateral step, a general increase in the requirements to walk could result in the crossover step being closer to the ceiling of stability, energy, and time requirements for the motor apparatus. Hence, we expected the effect of the swing leg on decisions to be stronger with ankle weights as compared to no additional weights (Fig. 2*D*).

#### Participants.

Forty-five participants from local universities were recruited, three of whom were excluded for violating the four-step criterion (>85.5%). This results in a sample size of *n* = 42 (mean age 21.8 yr, SD = 2.8; 24 females, 18 males; 40 right-handed, 2 with no data). All participants received €15.00 compensation and gave informed consent before starting the experiment.

#### Apparatus and stimuli.

The same apparatus and stimuli were used as in *experiment 1a*. However, we only included the 52.5° targets, which allowed us to increase the maximal distance from the starting line to the zone to 3.71 m. We replaced the obstacle of our previous experiments with a more prominent black protective grating (HWC-B34; height = 1.03 m, width = 0.5 m). To increase the weight of the legs, we strapped 2.5-kg ankle weights around each ankle.

#### Procedure.

The procedure was almost identical to *experiment 2*. However, instead of manipulating turning magnitudes and preview time, there were two blocks of walking with and without ankle weights. Ankle weights were applied or removed before the start of the respective block. Calibration of the starting line and time constraint was based on walking without ankle weights. Each participant completed a total of 6 familiarization trials and 84 experimental trials. Experimental trials included 2 (starting position: left/right foot at starting line) × 3 (point combination left/right: 40/60, 50/50, 60/40) with 7 trials for each unequal reward combination and 14 trials for the equal reward combination. Starting position and point combination were randomized between trials. The order of the blocks (weights or no weights) was counterbalanced between participants. Between blocks, participants had a 2-min pause to rest in a chair. The experiment lasted ∼75 min.

#### Data analysis.

We used the same offline and online analysis as in *experiment 1a*. After visual inspection, 4,347/5,040 (86.3%) trials were included in the statistical analysis. The remaining 13.7% of trials were predominantly excluded because participants made three or five steps instead of four (614 trials) and some trials because of various problems with the instruction or measurement (79 trials; see Supplemental Information for specifics).

For the model, we used the same priors, contrasts, and random effects as in *experiment 1b*, with turning magnitude being replaced by the factor weight (yes or no) as a centered sum contrast (−0.5 for no weights and +0.5 for weights). The formula for the model in the R script reads

$$\text{logit}\left({\text{p}}_{\text{Side}}\right)∼\text{Points}\_\text{R}*\text{Swing}\_\text{Leg}*\text{Weights}+\left(\text{Points}\_\text{R}*\text{Swing}\_\text{Leg}*\text{Weights}||\text{Subject}\right)$$

## RESULTS

### *Experiment 1a*: Is the Swing Leg Effect Moderated by Turning Magnitude?

**Figure 3.**
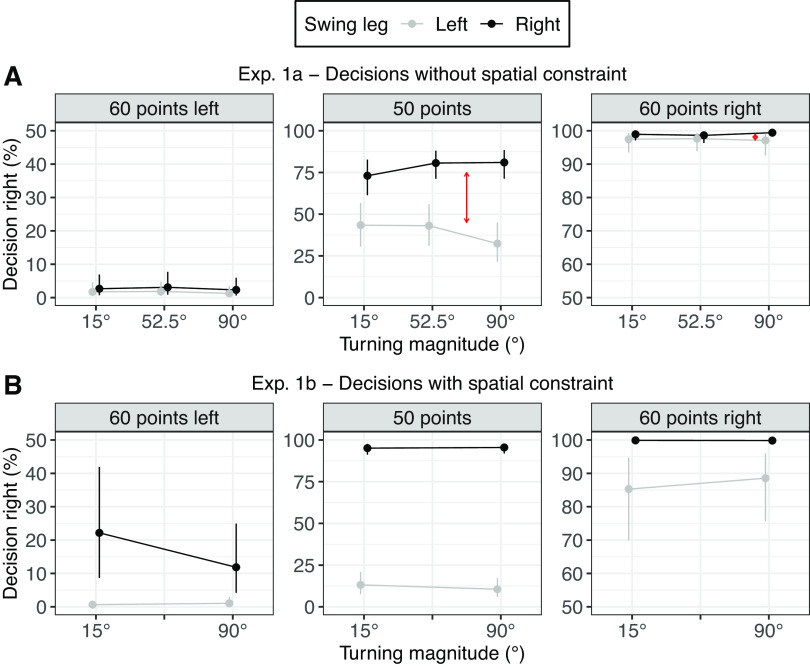
Effect of the swing leg in decision-making for different turning magnitudes and reward combinations. *A*: results for *experiment 1a*. The swing leg effect (SLE) was reliable for all reward combinations. Additionally, the SLE increased when comparing 52.5° to 90° for the equal reward combination (*center*, marked by red arrow) and in the 60 points right condition (*right*, marked by red arrow) but not reliable for the other conditions. *B*: results for *experiment 1b*. There was a generally stronger SLE compared to *experiment 1a*. However, there is reliable evidence that the SLE increased with turning magnitude. The *y*-axis displays the probability of walking toward the rightward side. 0% would mean that participants always went toward the left side; 100% means that participants always went toward the right side. Shown are the model estimates of the mean and 95% credible interval (CrI) for each condition. Note that the *y*-axis scale differs between equal (*center*) and unequal (*left* and *right*) reward conditions.

Tables 1 and 2 summarize the posterior distributions of *experiments 1a* and *1b*, respectively. Figure 3 displays the probability scales. Individual data for decision-making and the swing leg effect are displayed in Supplemental Figs. S1 and S2. Odds ratios below 1 correspond to a higher likelihood of walking toward the left side and odds ratios greater than 1 to the right side.

**Table 1.** Parameter summary of the fixed effects in experiment 1a

Effect	OR	95% CrI	*P*(OR < 1)
Intercept	1.27	[0.93 to 1.77]	0.063
**Unequal rewards**	**64.71**	**[25.15 to 168.55]**	**0.000**
Equal rewards	1.10	[0.98 to 1.26]	0.056
**Swing leg**	**0.33**	**[0.17 to 0.66]**	**0.999**
52.5° to 15°	1.08	[0.80 to 1.46]	0.310
90° to 52.5°	0.93	[0.72 to 1.20]	0.714
Unequal rewards: swing leg	0.77	[0.55 to 1.10]	0.928
**Equal rewards: swing leg**	**0.73**	**[0.60 to 0.87]**	**1.000**
Unequal rewards: 52.5° to 15°	0.89	[0.62 to 1.30]	0.731
Equal rewards: 52.5° to 15°	1.07	[0.92 to 1.26]	0.200
**Unequal rewards: 90° to 52.5°**	**1.42**	**[0.98 to 2.07]**	**0.031**
Equal rewards: 90° to 52.5°	0.93	[0.80 to 1.08]	0.830
Swing leg: 52.5° to 15°	0.94	[0.58 to 1.49]	0.610
**Swing leg: 90° to 52.5°**	**0.57**	**[0.33 to 0.98]**	**0.981**
Unequal rewards: swing leg: 52.5° to 15°	1.22	[0.67 to 2.20]	0.254
Equal rewards: swing leg: 52.5° to 15°	0.83	[0.62 to 1.10]	0.907
Unequal rewards: swing leg: 90° to 52.5°	0.60	[0.33 to 1.13]	0.945
Equal rewards: swing leg: 90° to 52.5°	1.03	[0.78 to 1.38]	0.407

Each parameter is summarized as the mean odds ratio (OR), the 95% credible interval (CrI), and the probability that the posterior is <1. Parameters with a high probability of being <1 or >1 are in bold (<0.05 or >0.95). For contrasts see methods. Model formula: $\text{logit}\left({\text{p}}_{\text{Side}}\right)∼\text{Points}\_\text{R}*\text{Swing}\_\text{Leg}*\text{Turning}\_\text{Magnitude}+\left(\text{Points}\_\text{R}*\text{Swing}\_\text{Leg}*\text{Turning}\_\text{Magnitude}||\text{Subject}\right)$.

**Table 2.** Parameter summary of the fixed effects in experiment 1b

Effect	OR	95% CrI	*P*(OR < 1)
**Intercept**	**1.71**	**[1.26 to 2.35]**	**0.001**
**Unequal rewards**	**50.04**	**[21.61 to 119.05]**	**0.000**
Equal rewards	0.99	[0.82 to 1.18]	0.561
**Swing leg**	**0.01**	**[0.01 to 0.03]**	**1.000**
90° to 15°	0.91	[0.58 to 1.42]	0.666
**Unequal rewards: swing leg**	**0.44**	**[0.21 to 0.88]**	**0.991**
**Equal rewards: swing leg**	**0.72**	**[0.50 to 1.01]**	**0.970**
Unequal rewards: 90° to 15°	1.03	[0.53 to 2.01]	0.467
Equal rewards: 90° to 15°	1.01	[0.77 to 1.32]	0.482
Swing leg: 90° to 15°	1.76	[0.69 to 4.56]	0.120
Unequal rewards: swing leg: 90° to 15°	0.76	[0.24 to 2.40]	0.678
**Equal rewards: swing leg: 90° to 15°**	**0.63**	**[0.38 to 1.05]**	**0.960**

Each parameter is summarized as the mean odds ratio (OR), the 95% credible interval (CrI), and the probability that the posterior is <1. Parameters with a high probability of being <1 or >1 are in bold (<0.05 or >0.95). For contrasts, see methods. Model formula: $\text{logit}\left({\text{p}}_{\text{Side}}\right)∼\text{Points}\_\text{R}*\text{Swing}\_\text{Leg}*\text{Turning}\_\text{Magnitude}+\left(\text{Points}\_\text{R}*\text{Swing}\_\text{Leg}*\text{Turning}\_\text{Magnitude}||\text{Subject}\right)$.

With regard to unequal reward combinations, participants almost always walked toward the side with higher rewards in *experiment 1a* (2.37% toward the right side when 60 points were on the left side, 98.53% toward the right side when 60 points were on the right side) and *experiment 1b* (3.68% toward the right side when 60 points were on the left side, 98.71% toward the right side when 60 points were on the right side). This indicates that the rewards were considered by the participants.

#### The swing leg effect was partially moderated by turning magnitude.

Participants’ decisions were biased by the swing leg after stepping into the central zone. Participants preferred walking toward the side enabling a lateral step, i.e., participants were less likely to walk toward the right target with a left swing leg compared to with a right swing leg [OR = 0.33, 95% CrI = 0.17 to 0.66, *P*(OR < 1) = 0.999]. The swing leg effect was greater for equal rewards (50/50) compared to unequal rewards [60/40 and 40/60; OR = 0.73, 95% CrI = 0.60 to 0.87, *P*(OR < 1) > 0.999]. Post hoc analysis indicated that even for unequal reward combinations, participants preferred walking toward the side enabling a lateral step [OR = 0.45, 95% CrI = 0.24 to 0.84, *P*(OR < 1) = 0.981]. However, because of the ceiling effects for unequal rewards, this swing leg effect is small on a probability scale (mean difference = 2.00%, 95% CrI = 0.40% to 5.37%).

The swing leg effect partially increased for larger turning magnitudes. The swing leg effect did not increase between the 15° and 52.5° targets [OR = 0.94, 95% CrI = 0.58 to 1.49, *P*(OR < 1) = 0.610]. However, the effect of the swing leg increased from 52.5° targets to 90° targets [OR = 0.57, 95% CrI = 0.33 to 0.98, *P*(OR < 1) = 0.981]. There was a tendency that reward combinations moderated the interaction between swing leg and angle for the latter angle comparison. The increase of the effect of the swing leg between 52.5° and 90° was slightly stronger in the 60/40 reward condition compared to the 40/60 reward condition [OR = 0.60, 95% CrI = 0.33 to 1.13, *P*(OR < 1) = 0.945], and the increase of the effect of the swing leg between 52.5° and 90° target angles was slightly stronger for the equal reward combination compared to the unequal reward combinations [OR = 0.83, 95% CrI = 0.62 to 1.10, *P*(OR < 1) = 0.907]. Because of this tendency, we ran post hoc comparisons for the interaction effect of swing leg and turning magnitude for all point combinations. For the 40/60 reward combination, there was no increase in the swing leg effect from 52.5° to 90° [OR = 0.91, 95% CrI = 0.45 to 2.83, *P*(OR < 1) = 0.572]. For the 60/40 reward combination, there was an increase of the swing leg effect between 52.5° and 90° [OR = 0.33, 95% CrI = 0.13 to 0.87, *P*(OR < 1) = 0.987], resulting possibly from a ceiling effect for 90° and a right swing leg, where participants only walked in very few trials toward the left side (see the 90° condition on *right* of plots in Fig. 3). For the equal reward combination, there was an increase of the swing leg effect between 52.5° and 90° [OR = 0.61, 95% CrI = 0.35 to 1.08 *P*(OR < 1) = 0.957].

In summary, the swing leg influenced participants’ choices. Participants preferred to walk toward the side enabling a lateral step, even when receiving less reward. Additionally, there was partial evidence for an increased swing leg effect for larger turning magnitudes.

#### Participants increasingly adapted their stepping strategy for larger turning magnitudes.

**Figure 4.**
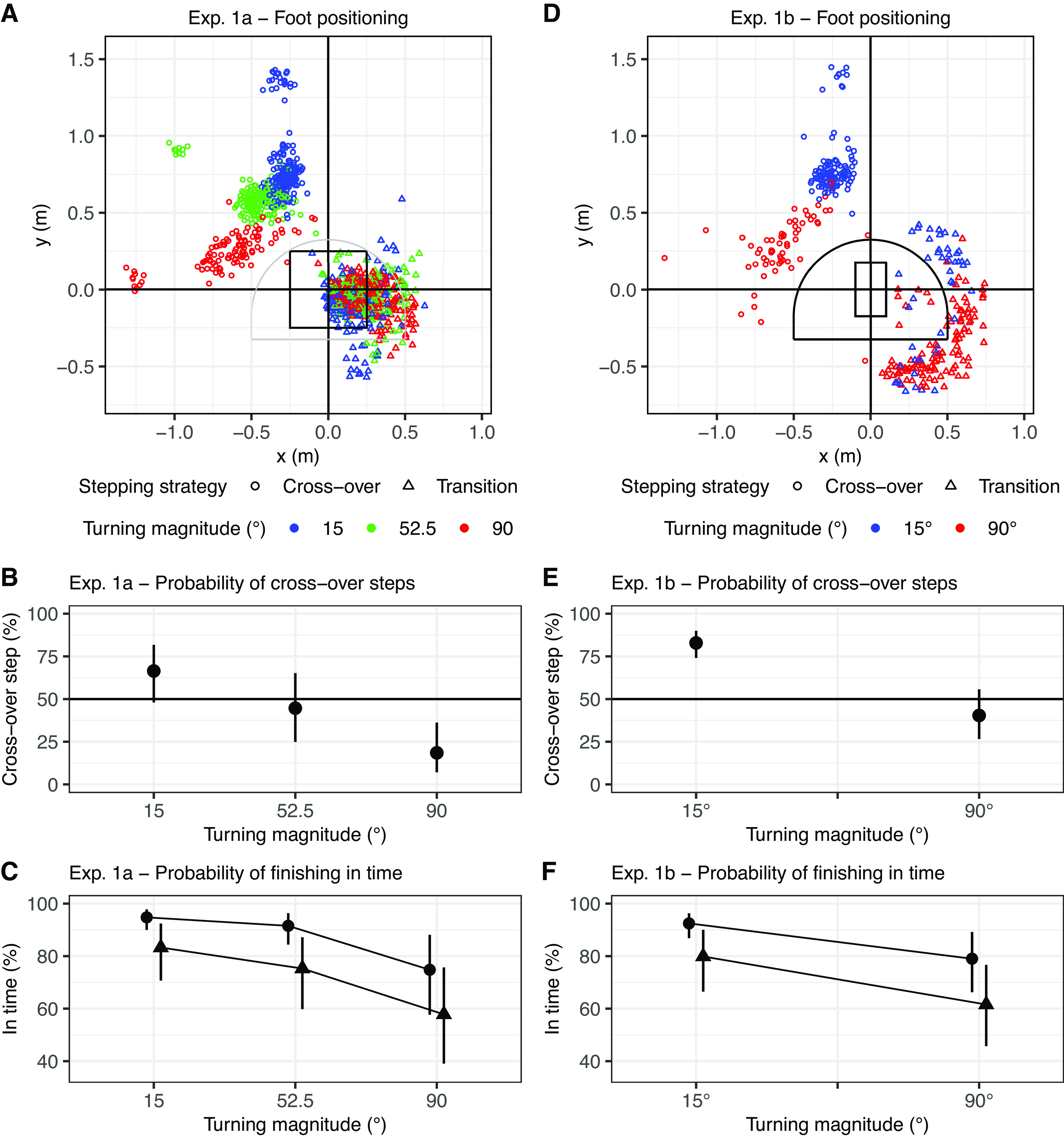
Characteristics of the step after reaching the zone and stepping strategies with varying turning magnitude. Only trials with decisions to walk toward the opposite side of the swing leg were analyzed (and presumably a crossover step was needed). *A*: position of the step after reaching the zone from the step in the zone in *experiment 1a*. Participants did not always cross the stance leg (circles, crossover steps) but stepped with the foot in the zone next to the stance leg (triangles, transition steps). The black rectangle represents the central zone. The gray semicircle represents the constraint area for the step after reaching the zone in *experiment 1b*. For brevity reasons, the figure shows only trials in which participants walked toward the left side with a right swing leg (hence blue). When participants walked toward the right side, the foot positioning was similar but mirrored. *B*: probability of a crossover step vs. a transition step for different turning magnitudes. Displayed are the mean estimate and 95% credible interval (CrI) on the response scale. Trials for both walking directions were analyzed. *C*: probability of finishing a trial in time for both stepping strategies. Trials with a transition step and larger turning magnitudes were more often too late compared to trials with a crossover step and smaller turning magnitudes. *D*: same as *A*, but for *experiment 1b*. Again, participants did not always perform crossover steps (circles) but still used transition steps (triangles). However, compared to *experiment 1a*, the transition step was now mostly outside the constraining carpet. *E*: same as *B*, but for *experiment 1b*. Compared to *experiment 1a*, participants more often made crossover steps, but the frequency of crossover steps still decreased with the turning magnitude. *F*: same as *C*, but for *experiment 1b*. Again, trials with transition steps and larger turning magnitudes were slower.

We originally assumed that a crossover step would be the predominantly observed stepping strategy when walking to the opposite side of the swing leg (19). To check this assumption, we analyzed the location of the step after reaching the zone (see Fig. 4; for additional methodological information, see Supplemental Information). A crossover step is marked by a position of the swing leg that crosses the stance leg. To our surprise, we observed that participants frequently avoided a crossover step but instead made a transition step (see Fig. 4*A*). That is, they placed both feet in the zone to enable a lateral step walking on.

The frequencies of crossover steps decreased for larger turning magnitudes, from 15° to 52.5° [OR = 0.40, 95% CrI = 0.27 to 0.59, *P*(OR < 1) > 0.999] and from 52.5° to 90° [OR = 0.26, 95% CrI = 0.15 to 0.44, *P*(OR < 1) > 0.999], or, in other words, the frequency of transition steps increased with turning magnitude. Participants infrequently made crossover steps for 90° targets (18.48%, 95% CrI = 7.11% to 36.23%; see Fig. 4*B*) and accordingly, they made predominantly a transition step.

The transition step takes an additional step to turn, and after seeing the results on stepping strategies, we hypothesized that the additional step could lead to a time cost. Consequently, we additionally analyzed whether the participants finished a trial within the required time constraint to receive the 60 points (see Fig. 4*C*). Indeed, trials with a transition step were slower compared to trials with a crossover step, leading to a higher probability of missing the time constraint and receiving the lower reward in *experiment 1a* [OR = 0.32, 95% CrI = 0.18 to 0.58, *P*(OR < 1) > 0.999]. Additionally, participants were slower and had a higher chance of missing the time constraint the larger the turning magnitude, from 15° to 52.5° [OR = 0.60, 95% CrI = 0.40 to 0.92, *P*(OR < 1) = 0.989] and from 52.5° to 90° [OR = 0.34, 95% CrI = 0.24 to 0.51, *P*(OR < 1) > 0.999]. Both effects additively lead to the lowest probability of being in time when making transition steps toward a 90° target (57.82%, 95% CrI = 39.07% to 75.70%). This suggests that participants made transition steps despite the cost of being too late and receiving less reward.

In sum, participants frequently avoided the crossover step and more so for larger turning magnitudes. Instead, they made a transition step, which could help to stabilize the turn but prolonged the turn duration. Albeit less efficient than a lateral step, the transition step likely absorbs some of the cost difference based on the current swing leg we aimed to manipulate in *experiment 1a*. To experimentally control the transition step, we constrained the foot placement in *experiment 1b*.

#### Repetition effect for stepping strategies for the equal reward combination.

For the equal reward combination, one could ask why participants made more costly crossover steps in the first place, given that there was no incentive to do so. One reason could be a repetition effect from the trial before often observed for task switching. To attend to possible repetition effects responsible for crossover steps in the equal reward condition, we additionally analyzed the influence of the side and the stepping strategy (crossing with a transition or a crossover step vs. lateral step) in the previous trial (*trial n − 1*) on the probability of crossing with a transition step or crossover step in the equal reward conditions of *trial n* in *experiment 1a* (see Supplemental Material, in particular Supplemental Fig. S4, for details). Repetition of the side only unreliably affected the frequency of crossing [OR = 1.13, 95% CrI = 0.95 to 1.36, *P*(OR < 1) = 0.088]. Additionally, there is a small but reliable effect of repeating the stepping strategy from the trial before [OR = 1.34, 95% CrI = 1.09 to 1.69, *P*(OR < 1) = 0.004]. That is, if participants made a crossover or transition step in the trial before, they were more likely to make a crossover or transition step in the trial afterward. There was no interaction with side repetition, suggesting that the repetition of the stepping strategy was independent of repeating the side. If true, this suggests carryover effects of a generalized action level (crossover/transition steps make crossover/transition steps more likely independent of the direction) on decision-making, providing further evidence that decision-making and action processes are directly intertwined.

Note, however, that participants still did make crossover or transition steps for equal rewards even if they did not repeat walking toward the previous side or made a lateral step previously (22.93%, 95% CrI = 16.35% to 30.36%). This means that other factors influence the occurrence of crossing behavior for equal rewards (e.g., attention or noise), which could be analyzed in future studies.

### *Experiment 1b*: Replication of *Experiment 1a* with a Stepping Constraint

Table 2 summarizes the posterior distribution, and Fig. 3*B* displays the model predictions on the probability scale. Individual data for decision-making and the swing leg effect are displayed in Supplemental Figs. S1 and S2 in the Supplemental Material.

Odds ratios < 1 correspond to a higher likelihood of walking toward the left side and odds ratios > 1 to the right side. With regard to unequal reward combinations, again participants almost always walked toward the side with higher rewards (3.68% toward the right side when 60 points were on the left side, 98.71% toward the right side when 60 points were on the right side). This indicates that the rewards were relevant for the participants.

#### Turning magnitude did not moderate the swing leg effect.

Participants again went less often toward the right target with a left swing leg compared to with a right swing leg [OR = 0.01, 95% CrI = 0.01 to 0.03, *P*(OR < 1) > 0.999]. Note that the effect of the swing leg was far stronger compared to *experiment 1a* (OR = 0.33). The effect of the swing leg again was greater for equal rewards (50/50) compared to unequal rewards [60/40 and 40/60; OR = 0.72, 95% CrI = 0.50 to 1.01, *P*(OR < 1) = 0.970]. There were also differences in the swing leg effect between unequal rewards. The swing leg effect increased for the left side (60 points left) compared to the right side (60 points right). This result suggests a higher preference for lateral steps when walking toward the left side. Post hoc analysis indicates that even for the weakest condition (60 points left) there was an effect of the swing leg [OR = 0.19, 95% CrI = 0.06 to 0.43, *P*(OR < 1) > 0.999]. On a probability scale, the difference for decision-making based on swing leg for unequal rewards was also greater in *experiment 1b* (mean difference = 14.1%, 95% CrI = 5.9% to 26.5%) compared to 2.00% in *experiment 1a*.

With regard to the interaction between swing leg in the zone and turning magnitude, the effect of the swing leg did not increase between the 15° and 90° targets [OR = 1.76, 95% CrI = 0.69 to 4.56, *P*(OR < 1) = 0.120]. The effect difference of the swing leg between 15° and 90° targets was greater for equal rewards compared to unequal rewards [OR = 0.63, 95% CrI = 0.38 to 1.05, *P*(OR < 1) = 0.960]. Post hoc analysis did not reveal a difference in the swing leg effect for the equal reward combination [OR = 0.71, 95% CrI = 0.26 to 1.84, *P*(OR < 1) = 0.761] or for the unequal reward combinations [OR = 2.78, 95% CrI = 0.79 to 10.11, *P*(OR < 1) = 0.060].

In summary, in both experiments, the swing leg influenced participants’ choices. Participants preferred to walk toward the side enabling a lateral step, even when receiving less reward. In *experiment 1a*, there was partial evidence for an increased swing leg effect for larger turning magnitudes. In *experiment 1b*, the size of the swing leg effect generally increased. However, no interaction was found between the swing leg effect and turning magnitude.

#### Participants more frequently made crossover steps but still adapted their stepping strategy.

The frequency of crossover steps increased for both turning magnitudes compared to *experiment 1a* [OR = 2.56, 95% CrI = 0.98 to 6.71, *P*(OR < 1) = 0.028; see Fig. 4*B*], with no interaction between them [OR = 0.95, 95% CrI = 0.42 to 2.11, *P*(OR < 1) = 0.557]. However, even with the step constraint on the floor participants still made transition steps (see Fig. 4*B*). For 90° targets, the probability of crossover steps versus transition steps was roughly even (mean estimated probability of crossover steps = 38.10%, 95% CrI = 20.31% to 58.45%). The transition steps were mostly placed outside the stepping constraint on the floor. Only in a few trials did participants step on the carpet area (46/786 trials). We did not remove these trials, as it would bias the estimations of the applied stepping strategies toward crossover steps.

The transition step was again slower compared to trials with a crossover step, leading to a higher chance of missing the time constraint and receiving the lower reward [OR = 0.37, 95% CrI = 0.21 to 0.64, *P*(OR < 1) > 0.999]. Participants were also slower and had a higher probability of missing the time constraint the larger the turning magnitude, from 15° to 90° [OR = 0.34, 95% CrI = 0.21 to 0.58, *P*(OR < 1) > 0.999]. Both effects additively led to the lowest probability of being in time when making transition steps toward a 90° target (61.58%, 95% CrI = 45.74% to 76.65%). Again, this suggests that participants made transition steps despite the cost of being too late and receiving less reward.

In sum, participants frequently avoided the crossover step and more so for larger turning magnitudes. Instead, they made a transition step, which could help to stabilize the turn but prolonged the turn duration. The effect of the swing leg became stronger with the spatial constraint on the floor in *experiment 1b*. But even though participants made crossover steps more frequently, participants’ preference for turning toward the side enabling a lateral step was not influenced by the turning magnitude.

### *Experiment 2*: Turning Magnitude Influences Decision-Making

#### Angle preference.

**Figure 5.**
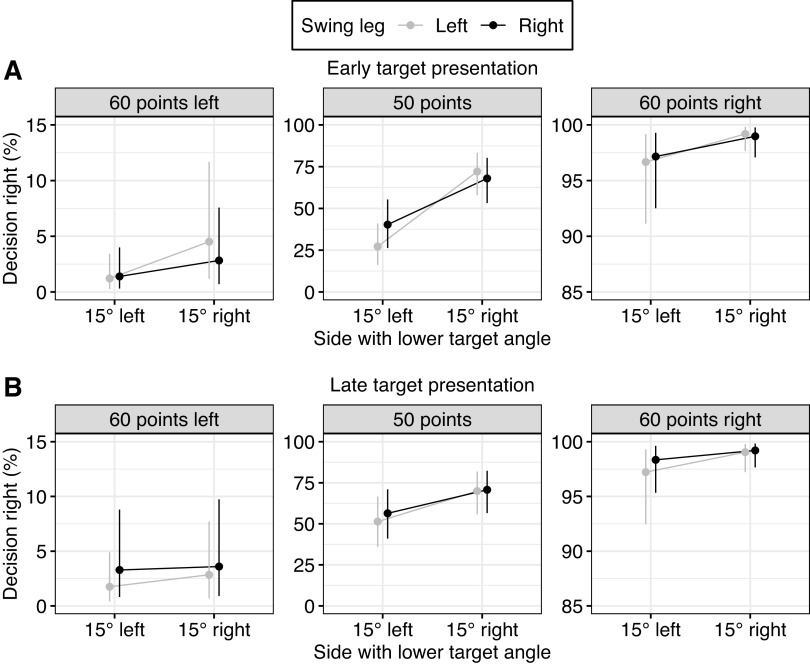
Effect of the turning magnitude and swing leg on decisions for different presentation timings of the targets and reward combinations in *experiment 2*. *A*: results when turning magnitudes were displayed before a trial started. *B*: results when turning magnitudes were displayed with reward while participants were walking. Only asymmetric angle combinations are included, meaning that 15° left indicates that the right turning magnitude was 90°. 0% means that participants always went toward the left side; 100% means that participants always went toward the right side. Lateral steps are realizable in the direction of the swing leg; hence, a swing leg effect would mean that participants went more often leftward given a left swing leg vs. a right swing leg (gray below black). A preference to walk toward the side with a smaller turning magnitude (15°) would be displayed by a positive slope between points. There was a reliable effect of turning magnitude: participants preferred to walk toward the side with a smaller turning magnitude. However, there was no reliable evidence for the swing leg effect (SLE) anymore. Displayed are the model estimates (probability scale) of the mean and 95% credible interval (CrI) for each condition. Note that the *y*-axis scale differs between equal (*center*) and unequal (*left* and *right*) reward conditions.

Table 3 summarizes the posterior distribution. Figure 5 displays the results on a probability scale. Individual data for decision-making and the swing leg effect are displayed in Supplemental Figs. S1 and S2 in the Supplemental Material. Odds ratios < 1 correspond to a higher likelihood of walking toward the left side and odds ratios > 1 to the right side.

**Table 3.** Parameter summary of the fixed effects in experiment 2

Effect	OR	95% CrI	*P*(OR < 1)
Intercept	1.31	[0.87 to 1.98]	0.095
Target timing	1.36	[0.94 to 1.97]	0.052
**Unequal rewards**	**61.80**	**[25.45 to 147.54]**	**0.000**
Equal rewards	1.02	[0.92 to 1.13]	0.368
Swing leg	0.86	[0.51 to 1.43]	0.725
**15° preference**	**2.67**	**[1.69 to 4.19]**	**0.000**
Unequal rewards: target timing	0.98	[0.53 to 1.78]	0.526
Equal rewards: target timing	1.07	[0.90 to 1.27]	0.226
Swing leg: target timing	0.73	[0.37 to 1.41]	0.833
Unequal rewards: swing leg	0.99	[0.66 to 1.49]	0.532
Equal rewards: swing leg	1.00	[0.83 to 1.21]	0.522
**15° preference: target timing**	**0.52**	**[0.29 to 0.97]**	**0.980**
Unequal rewards: 15° preference	1.23	[0.82 to 1.84]	0.146
Equal rewards: 15° preference	1.09	[0.90 to 1.33]	0.194
**Swing leg: 15° preference**	**1.60**	**[0.97 to 2.65]**	**0.034**
Unequal rewards: swing leg: target timing	1.12	[0.58 to 2.14]	0.366
Equal rewards: swing leg: target timing	1.22	[0.90 to 1.66]	0.099
Unequal rewards: 15° preference: target timing	1.24	[0.64 to 2.40]	0.259
Equal rewards: 15° preference: target timing	0.90	[0.63 to 1.31]	0.710
Unequal rewards: swing leg: 15° preference	0.74	[0.36 to 1.50]	0.797
Equal rewards: swing leg: 15° preference	0.93	[0.50 to 1.74]	0.592
Swing leg: asymmetric angle: target timing	1.01	[0.76 to 1.35]	0.470
Unequal rewards: swing leg: 15° preference: target timing	1.09	[0.47 to 2.50]	0.414
Equal rewards: swing leg: 15° preference: target timing	0.85	[0.53 to 1.35]	0.758

Each parameter is summarized as the mean odds ratio (OR), the 95% credible interval (CrI), and the probability that the posterior is <1. Parameters with a high probability of being <1 or >1 are in bold (<0.05 or >0.95). For contrasts, see methods. Model formula: $\text{logit}\left({\text{p}}_{\text{Side}}\right)∼\text{Target}\_\text{Timing}*\text{Points}\_\text{R}*\text{Swing}\_\text{Leg}*\text{Turning}\_\text{Magnitude}+\left(\text{Target}\_\text{Timing}*\text{Points}\_\text{R}*\text{Swing}\_\text{Leg}*\text{Turning}\_\text{Magnitude}||\text{Subject}\right)$.

Participants generally walked more frequently toward the 15° target compared to the 90° target [OR = 2.67, 95% CrI = 1.69 to 4.19, *P*(OR < 1) < 0.001]. The preference for the 15° targets was reduced when targets were presented late while participants were walking instead of early before the trial start [OR = 0.52, 95% CrI = 0.29 to 0.97, *P*(OR < 1) = 0.980]. Because the effect of the turning magnitude differed for reward combinations and for the timing of displaying the turning magnitudes, we made post hoc comparisons between turning magnitudes (15° left vs. 15° right) for the target timings individually. Participants preferred 15° targets when targets were presented early [OR = 3.69, 95% CrI = 2.14 to 6.32, *P*(OR < 1) < 0.001] but also when targets were presented late [OR = 1.92, 95% CrI = 1.12 to 3.35, *P*(OR < 1) = 0.009]. As in *experiment 1a*, participants almost always walked toward the side with higher rewards, and the effect of the turning magnitude is small on a probability scale for unequal reward combinations, especially for the late target presentation condition (mean difference = 1.2%, 95% CrI = 0.3% to 3.5%).

#### No swing leg effect for asymmetric turning magnitudes.

Although the focus was on comparing turning magnitudes in this study, we also analyzed the swing leg effect as in the previous study. It is noteworthy that an effect of the swing leg was unlikely in the model with only asymmetric turning magnitudes [OR = 0.86, 95% CrI = 0.51 to 1.43, *P*(OR < 1) = 0.725]. To follow up on this finding, we fitted another model that included the symmetric turning magnitudes to compare the effect of the swing leg between symmetric and asymmetric turning magnitudes. The effect of the swing leg indeed differed between asymmetric turning magnitudes and symmetric turning magnitudes [OR = 1.38, 95% CrI = 1.12 to 1.72, *P*(OR < 1) = 0.001]. As in *experiments 1a* and *1b*, participants preferred walking toward the side enabling a lateral step for symmetric turning magnitudes [OR = 0.65, 95% CrI = 0.47 to 0.90, *P*(OR < 1) = 0.995].

### *Experiment 3*. Swing Leg on Decision-Making with Ankle Weights

**Figure 6.**
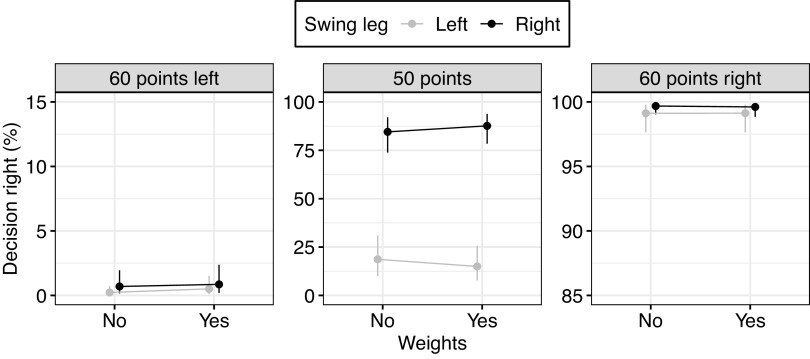
Effect of the swing leg on decision-making with and without ankle weights and for different reward combinations in *experiment 3*. The *x*-axis displays the probability of walking toward the rightward side. 0% would mean that participants always went toward the left side; 100% means that participants always went toward the right side. Lateral steps are realizable in the direction of the swing leg; hence, a swing leg effect would mean that participants more often went leftward given a left swing leg vs. a right swing leg (gray below black). The slope of the line between points indicates the influence of weights on the swing leg effect. A stronger swing leg effect with weights would be indicated by a divergence between both lines. There was no reliable evidence that the swing leg effect increased when participants wore ankle weights. Shown are the model mean estimates and 95% credible interval (CrI) for each condition. Note that the *y*-axis differs between (*center*) and unequal (*left* and *right*) reward conditions.

We analyzed the step into the zone for different reward combinations and ankle weights. Model estimations are displayed in Table 4, and a visual presentation of model estimates is displayed in Fig. 6. Individual data for decision-making and the swing leg effect are displayed in Supplemental Figs. S1 and S2 in the Supplemental Material.

**Table 4.** Parameter summary of the fixed effects in experiment 3

Effect	OR	95% CrI	*P*(OR < 1)
Intercept	1.01	[0.65 to 1.56]	0.475
**Unequal rewards**	**231.90**	**[94.57 to 587.35]**	**0.000**
Equal rewards	1.05	[0.87 to 1.25]	0.275
**Swing leg**	**0.17**	**[0.08 to 0.37]**	**1.000**
Weights	1.15	[0.76 to 1.72]	0.250
Unequal rewards: swing leg	0.94	[0.52 to 1.71]	0.577
**Equal rewards: swing leg**	**0.42**	**[0.27 to 0.65]**	**1.000**
Unequal rewards: weights	0.74	[0.42 to 1.29]	0.862
Equal rewards: weights	0.93	[0.74 to 1.17]	0.729
Swing leg: weights	1.10	[0.54 to 2.18]	0.392
Unequal rewards: swing leg: weights	0.84	[0.38 to 1.86]	0.674
Equal rewards: swing leg: eights	0.73	[0.47 to 1.13]	0.922

Each parameter is summarized as the mean odds ratio (OR), the 95% credible interval (CrI), and the probability that the posterior is <1. Parameters with a high probability of being <1 or >1 are in bold (<0.05 or >0.95). For contrasts, see methods. Model formula: $\text{logit}\left({\text{p}}_{\text{Side}}\right)∼\text{Points}\_\text{R}*\text{Swing}\_\text{Leg}*\text{Weights}+\left(\text{Points}\_\text{R}*\text{Swing}\_\text{Leg}*\text{Weights}||\text{Subject}\right)$.

#### Swing leg influenced decisions independent of ankle weights.

In regard to participants’ choices, participants went less often toward the left target with a right swing leg in the zone compared to a left swing leg [OR = 0.17, 95% CrI = 0.08 to 0.37, *P*(OR < 1) > 0.999]. The effect of the swing leg lies in between *experiment 1a* (OR = 0.33) and *experiment 1b* (OR = 0.01). The effect of the swing leg was again greater for equal rewards (50/50) compared to unequal rewards [60/40 and 40/60; OR = 0.42, 95% CrI = 0.27 to 0.65, *P*(OR < 1) > 0.999]. Post hoc analysis showed a swing leg effect even for unequal rewards [OR = 0.40, 95% CrI = 0.15 to 1.08, *P*(OR < 1) > 0.965]. On a probability scale, the difference for decision-making based on swing leg was small as in *experiment 1* (mean difference = 1.0%, 95% CrI = 0.2% to 2.6%).

The effect of the swing leg did not increase when participants wore ankle weights [OR = 1.10, 95% CrI = 0.54 to 2.18, *P*(OR < 1) = 0.392]. There was only a tendency that weights differently affected the swing leg effect for equal and unequal rewards. The effect of weights on the swing leg effect was tendentially stronger with equal rewards [OR = 0.73, 95% CrI = 0.47 to 1.13, *P*(OR < 1) =0.922]. For equal rewards, there was a tendency that weights increased the swing leg effect [OR = 0.58, 95% CrI = 0.26 to 1.32, *P*(OR < 1) =0.901].

We ran an additional analysis for the potential time cost of weights. The ankle weights increased the time needed to finish a trial independent of the swing leg [$\hat{\beta }$ = 0.10 s, 95% CrI = 0.06 s to 0.14 s, *P*($\hat{\beta }$ < 0) < 0.001]. An unpreferred swing leg requiring a crossover step had a similar effect on the time needed to finish the trial {[$\hat{\beta }$ = 0.11 s, 95% CrI = 0.07 s to 0.15 s, *P*($\hat{\beta }$ < 0) < 0.001]}.

In summary, we replicated the effect of the swing leg. Ankle weights decreased the time margin to finish a trial independent of the swing leg, i.e., required stepping strategy. But ankle weights did not robustly influence the effect of the swing leg.

## DISCUSSION

### *Experiment 1a* and *Experiment 1b*

We argued that the motor cost of turning depends on the current swing leg and that this cost difference is moderated by turning magnitude. If action cost influences participants’ decisions, the preference for lateral steps should also be moderated by turning magnitude.

In both experiments participants indeed preferred walking toward the side enabling a lateral step, that is, they showed a swing leg effect. In *experiment 1a*, the swing leg effect was largest for the highest turning magnitude. When the stepping behavior was further constrained in *experiment 1b*, the swing leg effect generally increased; however, it no longer depended on the turning magnitude. Additionally, in *experiment 1b* participants tended less frequently to substitute crossover steps with transition steps.

Overall, the finding that participants preferred walking toward the side enabling a lateral step replicated the findings of our previous study (14), thereby providing further support for the impact of concurrent movement on decision-making (13, 15, 39). Going beyond this replication, we predicted that concurrent actions influence decision-making by the emerging cost dynamics and not (only) by cognitive cross talk (15). Our results support this hypothesis, as changes in motor costs were reflected in decision-making. Specifically, this is evidenced by, first, an increased swing leg effect for larger turning requirements in *experiment 1a* and, second, a larger swing leg effect in *experiment 1b* compared to *experiment 1a*. The larger swing leg effect in *experiment 1b* could be a result of the additional foot placement and orientation constraints given the smaller central zone (see Supplemental Information). Foot placement and orientation are important for the adaptation of mediolateral stability of walking (40, 41). Hence, crossover steps could have become generally more costly with the spatial constraint, thereby indicating that the swing leg effect is moderated by action costs.

However, not all variations in motor costs were reflected in decision-making. For instance, there was no interaction between turning magnitude and swing leg in *experiment 1b*. One explanation for this lack of an interaction could be the additional task constraint. In research on reaching, for example, it has been suggested that with more task constraints it is increasingly difficult to meet all the requirements of a given task and succeed in it while still attending to motor costs. Consequently, it has been argued that the influence of more subtle motor cost differences on decision-making decreases (21). Another second explanation for the missing interaction could reside in the transition step. The transition step affords a “neutral” body state, in which the more stable lateral step can be made in either direction. Hence the motor cost differences between the turning directions would be reduced. Accordingly, the increased number of transition steps for larger turning magnitudes could indicate that crossover steps indeed became more costly and were therefore replaced with transition steps. If this is true, then, instead of compromising reward-based decisions, motor cost differences were counteracted by adapting the stepping behavior.

When analyzing why participants crossed for equal reward combinations, we additionally observed repetition of the stepping strategy (crossing with a transition step or crossover step vs. lateral step) independent of a side repetition for the equal reward combination in *experiment 1a*. If true, this suggests carryover effects of an abstract action-level representation on decision-making, providing further evidence that decision-making and action processes are intertwined. From the perspective that decision-making includes competition between action representations in fronto-parietal areas (42), it could suggest that these abstract action features have a higher baseline activation for a short time after the trial, leading to the increased likelihood of activation and therefore a decision to repeat the motor behavior. These repetition effects could open a window to scrutinize elements of decision-making on different hierarchical levels of the decision process and provide an opportunity for future research.

Together, the larger swing leg effect in *experiment 1b* compared to *experiment 1a* and the increased swing leg effect for larger turning magnitudes in *experiment 1a* provide additional evidence that motor costs influence decisions with concurrent movement. Furthermore, it seems that motor cost differences can be overcome by adapting concurrent movement.

### *Experiment 2*: Turning Magnitude Influences Decision-Making

Concerning the turning magnitude, participants preferred targets with a smaller angle compared to targets with a larger turning magnitude, even at the expense of receiving less reward. The effect of turning magnitude was observable when turning magnitudes were presented late while participants were walking, although weaker. As the energetic demands increase with turning magnitude (3, 4), this could suggest that participants integrate the cost differences between asymmetric turn decisions while walking. A similar preference to move toward targets with a smaller angle was observed in some dynamic reaching experiments (26, 34). Our results extend these findings to walking.

Regarding the swing leg effect, participants again preferred walking toward the side enabling a lateral step for symmetric turning magnitudes, as in *experiment 1a* and *experiment 1b*. However, we did not find evidence for a swing leg effect with asymmetric turning magnitudes (e.g., 15° left vs. 90° right). Possible reasons for the absence of the swing leg effect with asymmetric turning magnitudes are discussed in the *General Discussion*.

This experiment was the first in which motor costs were influenced by an environmental manipulation independent of the body state. In contrast to the body state, the cost differences from the environment were random and not predictable when presented late while participants were walking. Even for late presentations, the influence of turning magnitude suggests that environmental cost differences can be integrated on the fly without long-term anticipation (1). Additionally, the preference for 15° targets was stronger when turning magnitudes were presented before the trial, indicating that participants utilized the early target presentation to weigh options based on their costs. One mechanism behind this result could be competition on the level of action representations, which increases dynamically with time of presentation (6), leading to a stronger activation and bias for the 15° target even before reward options are displayed.

### *Experiment 3*: Swing Leg on Decision-Making with Ankle Weights

When adding ankle weights, we observed that the time to finish increased independent of the required stepping strategy. Even with the increase in time, the swing leg effect remained and was unaffected by the presence of the ankle weights, replicating the finding of the previous experiments and extending it to when the body state is manipulated by additional ankle weights.

### General Discussion

Embodied decision accounts argue that the dynamic changes in the motor costs of behavioral options influence decision-making. Indeed, this claim has received empirical support (12, 14, 15, 17). However, it is unclear to what extent this truly reflects the impact of dynamic motor costs (15). Here, we addressed this question by systematically manipulating the motor costs of choices during concurrent movement.

To briefly summarize our main findings, in *experiment 1a* and *experiment 1b* we manipulated the motor costs of crossover steps compared to lateral steps during walking by symmetrically increasing the required turning magnitude. Participants generally preferred walking toward the side enabling a lateral step, thereby showing what we refer to as the swing leg effect. In *experiment 1a*, the swing leg effect was larger for higher turning magnitudes. When the stepping behavior was additionally constrained in *experiment 1b*, the swing leg effect further increased, albeit no longer being modulated by the turning magnitude. Additionally, when we investigated participants’ stepping behavior, participants less frequently substituted crossover steps with transition steps. In *experiment 2*, we manipulated motor costs on top of the required stepping strategy by increasing the required turning magnitude asymmetrically. When choices required asymmetric turns, the swing leg effect disappeared. Instead, only the turning magnitude itself influenced participants’ decisions. The participants preferred walking toward targets requiring smaller turning angles. Finally, in *experiment 3*, we manipulated the motor costs of crossover steps compared to lateral steps by adding weights to the ankles. Results revealed that participants showed a swing leg effect independent of weights. Together, the emergence of the swing leg effect under almost all conditions and across the three experiments replicates and supports the earlier studies showing that concurrent movement can influence decision-making (13–15). These findings generally support claims of action-based models for which action execution is an integral part of the decision process (8).

Next to replicating the influence of concurrent movement on decision-making, the finding that changes in motor costs were reflected in decision-making supports the claim that concurrent actions influence decision-making by the emerging cost dynamics and not (only) by cognitive cross talk (15). This concerns the increased swing leg effect for larger turning requirements in *experiment 1a*, the larger swing leg effect in *experiment 1b* compared to *experiment 1a*, and the influence of turning magnitude when displayed concurrently with movement execution in *experiment 2*. These findings add to the influence of motor costs on decision-making choices without concurrent action (1, 43, 44). For such sequential decisions, time and force have been identified as cost dimensions (45). When walking, relevant cost dimensions include motor variables like stability, forward progression, muscle torque, time, or energetic considerations (18, 45, 46). A challenge for future studies could be to disentangle these cost dimensions and their influence on decision-making while moving.

Besides the influence of motor costs on reward-based decisions, the increased rate of transition steps for larger turning magnitudes in *experiment 1a* and *experiment 1b* also suggests that the motor costs led to adaptations in concurrent motor control. The adaptation of motor control may in turn allow overcoming cost differences between choices. This close interaction between “continuous motor decisions” and “discrete reward-based decisions” (10) also highlights the reciprocal influence between these processes as proposed by action-based models (5, 8, 47).

However, some variations in motor costs were not reflected in decision-making. This concerns the missing interaction between turning magnitude and swing leg in *experiment 1b*, the missing swing leg effect in *experiment 2*, and the missing moderation of the swing leg effect by ankle weights in *experiment 3*. In this regard, *experiment 2* is especially interesting, as asymmetric targets provided an additional cost dimension besides the swing leg. That is, the side of the lateral step was independent of the side with the smaller turning magnitude over trials, and, consequently, both should influence decision-making. One reason why variations in motor costs are not reflected in decision-making could be limitations in fully integrating motor costs during movement execution. Such missing or suboptimal integration of motor costs has been reported in other dynamic decision tasks with concurrent movement (26, 48). It is further conceivable that temporal restrictions and the dynamic nature of costs impose limits on estimating motor costs concurrent with movement (7, 8). If this is true, future studies could focus on the integration of action costs from multiple sources, with different time constraints, or in comparison with sequential decisions.

In conclusion, the decision of whether you should dribble and pass to your opponent to the left or the right depends on the motor cost dynamics while approaching the opponent. In such dynamic situations, motor costs appear to influence both the level of decision-making and the level of motor control, highlighting the reciprocal relationship between motor cost dynamics and decision-making as suggested by models of embodied decision-making (5, 8, 47).

## DATA AVAILABILITY

The data, custom codes for the statistical analysis, and additional material are available at https://doi.org/10.17605/OSF.IO/C8MUS.

## GRANTS

This work was supported by the German Research Foundation (DFG) with two grants awarded to R.C.-B. (CA 635/4-1) and O.H. (HE 6710/4-1).

## DISCLAIMERS

The funders had no role in the study design, data collection, analysis, decision to publish, or preparation of the manuscript.

## DISCLOSURES

No conflicts of interest, financial or otherwise, are declared by the authors.

## AUTHOR CONTRIBUTIONS

E.G., O.H., and R.C.-B. conceived and designed research; E.G. performed experiments; E.G. analyzed data; E.G., O.H., and R.C.-B. interpreted results of experiments; E.G. prepared figures; E.G. drafted manuscript; E.G., P.R., O.H., and R.C.-B. edited and revised manuscript; E.G., P.R., O.H., and R.C.-B. approved final version of manuscript.

## ENDNOTE

At the request of the authors, readers are herein alerted to the fact that additional materials related to this manuscript may be found at https://doi.org/10.17605/OSF.IO/C8MUS. These materials are not a part of this manuscript and have not undergone peer review by the American Physiological Society (APS). APS and the journal editors take no responsibility for these materials, for the website address, or for any links to or from it.

## References

[1] Cos I, Duque J, Cisek P. Rapid prediction of biomechanical costs during action decisions. J Neurophysiol 112: 1256–1266, 2014. doi:10.1152/jn.00147.2014. 24899673

[2] Hartmann MN, Hager OM, Tobler PN, Kaiser S. Parabolic discounting of monetary rewards by physical effort. Behav Processes 100: 192–196, 2013. doi:10.1016/j.beproc.2013.09.014. 24140077

[3] Wilson RP, Griffiths IW, Legg PA, Friswell MI, Bidder OR, Halsey LG, Lambertucci SA, Shepard EL. Turn costs change the value of animal search paths. Ecol Lett 16: 1145–1150, 2013. doi:10.1111/ele.12149. 23848530

[4] McNarry MA, Wilson RP, Holton MD, Griffiths IW, Mackintosh KA. Investigating the relationship between energy expenditure, walking speed and angle of turning in humans. PLoS One 12: e0182333, 2017. doi:10.1371/journal.pone.0182333. 28796796 PMC5552125

[5] Lepora NF, Pezzulo G. Embodied choice: how action influences perceptual decision making. PLoS Comput Biol 11: e1004110, 2015. doi:10.1371/journal.pcbi.1004110. 25849349 PMC4388485

[6] Cisek P. Cortical mechanisms of action selection: the affordance competition hypothesis. Philos Trans R Soc Lond B Biol Sci 362: 1585–1599, 2007. doi:10.1098/rstb.2007.2054. 17428779 PMC2440773

[7] Newell A, Simon HA. Human Problem Solving. Oxford, UK: Prentice-Hall, 1972.

[8] Wispinski NJ, Gallivan JP, Chapman CS. Models, movements, and minds: bridging the gap between decision making and action. Ann NY Acad Sci 1464: 30–51, 2020. doi:10.1111/nyas.13973. 30312476

[9] Todorov E, Jordan MI. Optimal feedback control as a theory of motor coordination. Nat Neurosci 5: 1226–1235, 2002. doi:10.1038/nn963. 12404008

[10] Yoo SB, Hayden BY, Pearson JM. Continuous decisions. Philos Trans R Soc Lond B Biol Sci 376: 20190664, 2021. doi:10.1098/rstb.2019.0664. 33423634 PMC7815426

[11] Gordon J, Maselli A, Lancia GL, Thiery T, Cisek P, Pezzulo G. The road towards understanding embodied decisions. Neurosci Biobehav Rev 131: 722–736, 2021. doi:10.1016/j.neubiorev.2021.09.034. 34563562 PMC7614807

[12] Nashed JY, Crevecoeur F, Scott SH. Rapid online selection between multiple motor plans. J Neurosci 34: 1769–1780, 2014. doi:10.1523/JNEUROSCI.3063-13.2014. 24478359 PMC8186509

[13] Marti-Marca A, Deco G, Cos I. Visual-reward driven changes of movement during action execution. Sci Rep 10: 15527, 2020. doi:10.1038/s41598-020-72220-2. 32968102 PMC7511350

[14] Grießbach E, Incagli F, Herbort O, Cañal-Bruland R. Body dynamics of gait affect value-based decisions. Sci Rep 11: 11894, 2021. doi:10.1038/s41598-021-91285-1. 34088941 PMC8178314

[15] Raßbach P, Grießbach E, Cañal-Bruland R, Herbort O. Deciding while moving: cognitive interference biases value-based decisions. Acta Psychol (Amst) 221: 103449, 2021. doi:10.1016/j.actpsy.2021.103449. 34801882

[16] Rangel A, Hare T. Neural computations associated with goal-directed choice. Curr Opin Neurobiol 20: 262–270, 2010. doi:10.1016/j.conb.2010.03.001. 20338744

[17] Cos I, Pezzulo G, Cisek P. Changes of mind after movement onset depend on the state of the motor system. eNeuro 8: ENEURO.0174-21.2021, 2021. doi:10.1523/ENEURO.0174-21.2021. 34772692 PMC8675088

[18] Moraes R, Allard F, Patla AE. Validating determinants for an alternate foot placement selection algorithm during human locomotion in cluttered terrain. J Neurophysiol 98: 1928–1940, 2007. doi:10.1152/jn.00044.2006. 17686917

[19] Taylor MJ, Dabnichki P, Strike SC. A three-dimensional biomechanical comparison between turning strategies during the stance phase of walking. Hum Mov Sci 24: 558–573, 2005. doi:10.1016/j.humov.2005.07.005. 16129503

[20] He C, Xu R, Zhao M, Guo Y, Jiang S, He F, Ming D. Dynamic stability and spatiotemporal parameters during turning in healthy young adults. Biomed Eng Online 17: 127, 2018. doi:10.1186/s12938-018-0558-5. 30241535 PMC6151057

[21] Akram SB, Frank JS, Chenouri S. Turning behavior in healthy older adults: is there a preference for step versus spin turns? Gait Posture 31: 23–26, 2010. doi:10.1016/j.gaitpost.2009.08.238. 19765996

[22] Patla AE, Prentice SD, Robinson C, Neufeld J. Visual control of locomotion: strategies for changing direction and for going over obstacles. J Exp Psychol Hum Percept Perform 17: 603–634, 1991. doi:10.1037//0096-1523.17.3.603. 1834781

[23] Hommel B. Automatic stimulus-response translation in dual-task performance. J Exp Psychol Hum Percept Perform 24: 1368–1384, 1998. doi:10.1037//0096-1523.24.5.1368. 9988597

[24] Janczyk M, Pfister R, Hommel B, Kunde W. Who is talking in backward crosstalk? Disentangling response- from goal-conflict in dual-task performance. Cognition 132: 30–43, 2014. doi:10.1016/j.cognition.2014.03.001. 24747873

[25] Aczel B, Szollosi A, Palfi B, Szaszi B, Kieslich PJ. Is action execution part of the decision-making process? An investigation of the embodied choice hypothesis. J Exp Psychol Learn Mem Cogn 44: 918–926, 2018. doi:10.1037/xlm0000484. 29400481

[26] Michalski J, Green AM, Cisek P. Reaching decisions during ongoing movements. J Neurophysiol 122: 1090–1102, 2020. doi:10.1152/jn.00613.2019. 32049585 PMC7099481

[27] Banks JJ, Chang WR, Xu X, Chang CC. Using horizontal heel displacement to identify heel strike instants in normal gait. Gait Posture 42: 101–103, 2015. doi:10.1016/j.gaitpost.2015.03.015. 25907129

[28] R Core Team. R: A Language and Environment for Statistical Computing. Vienna, Austria: R Foundation for Statistical Computing, 2019.

[29] Bürkner PC. brms: An R package for Bayesian multilevel models using Stan. J Stat Softw 80: 1–28, 2017. doi:10.18637/jss.v080.i01.

[30] Kruschke JK. Bayesian analysis reporting guidelines. Nat Hum Behav 5: 1282–1291, 2021. doi:10.1038/s41562-021-01177-7. 34400814 PMC8526359

[31] Schad DJ, Vasishth S, Hohenstein S, Kliegl R. How to capitalize on a priori contrasts in linear (mixed) models: a tutorial. J Mem Lang 110: 104038, 2020. doi:10.1016/j.jml.2019.104038.

[32] Barr DJ, Levy R, Scheepers C, Tily HJ. Random effects structure for confirmatory hypothesis testing: keep it maximal. J Mem Lang 68: 255–278, 2013. doi:10.1016/j.jml.2012.11.001. PMC3881361 24403724

[33] Oberauer K. The importance of random slopes in mixed models for Bayesian hypothesis testing. Psychol Sci 33: 648–665, 2022. doi:10.1177/09567976211046884. 35357978

[34] Hesse C, Kangur K, Hunt AR. Decision making in slow and rapid reaching: sacrificing success to minimize effort. Cognition 205: 104426, 2020. doi:10.1016/j.cognition.2020.104426. 32800570

[35] Russell DM, Haworth JL, Martinez-Garza C. Coordination dynamics of (a)symmetrically loaded gait. Exp Brain Res 234: 867–881, 2016. doi:10.1007/s00221-015-4512-5. 26661338

[36] Graves JE, Martin AD, Miltenberger LA, Pollock ML. Physiological responses to walking with hand weights, wrist weights, and ankle weights. Med Sci Sports Exerc 20: 265–271, 1988. doi:10.1249/00005768-198806000-00009. 3386506

[37] Skinner HB, Barrack RL. Ankle weighting effect on gait in able-bodied adults. Arch Phys Med Rehabil 71: 112–115, 1990. 2105707

[38] Werth J, Bohm S, Klenk J, König M, Sczuka KS, Schroll A, Epro G, Mandla-Liebsch M, Rapp K, Potthast W, Arampatzis A, Karamanidis K. Stability recovery performance in adults over a wide age range: a multicentre reliability analysis using different lean-and-release test protocols. J Biomech 125: 110584, 2021. doi:10.1016/j.jbiomech.2021.110584. 34217031

[39] Burk D, Ingram JN, Franklin DW, Shadlen MN, Wolpert DM. Motor effort alters changes of mind in sensorimotor decision making. PLoS One 9: e92681, 2014. doi:10.1371/journal.pone.0092681. 24651615 PMC3961398

[40] van Leeuwen AM, van Dieën JH, Daffertshofer A, Bruijn SM. Active foot placement control ensures stable gait: effect of constraints on foot placement and ankle moments. PLoS One 15: e0242215, 2020. doi:10.1371/journal.pone.0242215. 33332421 PMC7746185

[41] Rebula JR, Ojeda LV, Adamczyk PG, Kuo AD. The stabilizing properties of foot yaw in human walking. J Biomech 53: 1–8, 2017. doi:10.1016/j.jbiomech.2016.11.059. 28161109 PMC6311129

[42] Cisek P. Making decisions through a distributed consensus. Curr Opin Neurobiol 22: 927–936, 2012. doi:10.1016/j.conb.2012.05.007. 22683275

[43] Hagura N, Haggard P, Diedrichsen J. Perceptual decisions are biased by the cost to act. Elife 6: e18422, 2017. doi:10.7554/eLife.18422. 28219479 PMC5319835

[44] Pierrieau E, Lepage JF, Bernier PM. Action costs rapidly and automatically interfere with reward-based decision-making in a reaching task. eNeuro 8: ENEURO.0247-21.2021, 2021. doi:10.1523/ENEURO.0247-21.2021. 34281978 PMC8354712

[45] Morel P, Ulbrich P, Gail A. What makes a reach movement effortful? Physical effort discounting supports common minimization principles in decision making and motor control. PLoS Biol 15: e2001323–e2001323, 2017. doi:10.1371/journal.pbio.2001323. 28586347 PMC5460791

[46] Minetti AE, Ardigò LP, Saibene F. The transition between walking and running in humans—metabolic and mechanical aspects at different gradients. Acta Physiol Scand 150: 315–323, 1994. doi:10.1111/j.1748-1716.1994.tb09692.x. 8010138

[47] Cisek P, Kalaska JF. Neural mechanisms for interacting with a world full of action choices. Annu Rev Neurosci 33: 269–298, 2010. doi:10.1146/annurev.neuro.051508.135409. 20345247

[48] Bakker RS, Weijer RH, van Beers RJ, Selen LP, Medendorp WP. Decisions in motion: passive body acceleration modulates hand choice. J Neurophysiol 117: 2250–2261, 2017. doi:10.1152/jn.00022.2017. 28250146 PMC5461666

